# Polyphenols and Other Bioactive Compounds of *Sideritis* Plants and Their Potential Biological Activity

**DOI:** 10.3390/molecules25163763

**Published:** 2020-08-18

**Authors:** Dorota Żyżelewicz, Kamila Kulbat-Warycha, Joanna Oracz, Kacper Żyżelewicz

**Affiliations:** 1 Institute of Food Technology and Analysis, Faculty of Biotechnology and Food Sciences, Lodz University of Technology, 90-924 Lodz, Poland; kamila.kulbat-warycha@p.lodz.pl (K.K.-W.); joanna.oracz@p.lodz.pl (J.O.); 2 Faculty of Health Sciences, Medical University of Lodz, 90-647 Lodz, Poland; kaczyze@gmail.com

**Keywords:** *Sideritis scardica*, polyphenols, biological active compounds, gut microbiota, antioxidant, anti-inflammatory and neuroprotective properties

## Abstract

Due to the growing problem of obesity associated with type 2 diabetes and cardiovascular diseases, causes of obesity are extensively investigated. In addition to a high caloric diet and low physical activity, gut microbiota disturbance may have a potential impact on excessive weight gain. Some reports indicate differences in the composition of the intestinal microflora of obese people in comparison to lean. Bioactive compounds of natural origin with beneficial and multifaceted effects on the body are more frequently used in prevention and treatment of many metabolic diseases including obesity. *Sideritis scardica* is traditionally consumed as mountain tea in the Balkans to strengthen the body and improve mood. Many reports indicate a positive effect on digestive system, weight loss, and prevention of insulin resistance. Additionally, it exhibits antioxidant activity and anti-inflammatory effects. The positive effect of *Sideritis scardica* extracts on memory and general cognitive abilities is indicated as well. The multilevel positive effect on the body appears to originate from the abundant occurrence of phenolic compounds, especially phenolic acids in *Sideritis scardica* extracts. However, mechanisms underlying their action require careful discussion and further research. Therefore, the objective of this review is to summarize the available knowledge on the role and mechanism of action of biologically active compounds of *Sideritis scardica* and other related species from the genus *Sideritis*.

## 1. Introduction

Plants from the genus *Sideritis* (belongs to the *Lamiaceae* family) occur mainly in the Mediterranean area, on the islands of Macaronesia and in western and central Asia [1]. The genus *Sideritis* includes more than 150 species of annual or perennial xerophytic and thermophytic plants naturally growing in mountain areas. From ancient times *Sideritis* plants have been used in folk medicine, most often in the form of aromatic herbal tea [2,3,4].

*Sideritis scardica* (known as Ironwort, “Olympus tea”, “Pirin Tea”, or “Mursalski Tea”) is traditionally used in the Balkans and the Middle East for the preparation of herbal tea for strengthening the body and improving the mood. In Bulgaria it is considered as an aphrodisiac. It is a xerophytic perennial herb growing in the mountains at high altitudes over 1000 m a.s.l. endemically on the Balkan Peninsula [2,3].

In folk medicine it has been used for ages for the treatment of inflammation of different origin, especially in the run of common cold, asthma, bronchitis, and gastrointestinal disorders. It was believed that drinking Olympus tea relieves pain, also rheumatic, as well as reduces stress and anxiety. Not only the *S. scardica* but also other species of the genus *Sideritis* have been used for centuries because of their anti-inflammatory, antimicrobial, and anxiolytic properties. The name for the plant derives from the Greek word “σίδηρος” and can be literally translated as “iron”, as in ancient times *Sideritis* was used as a valuable medicine for soldiers in healing wounds from iron weapons [4,5].

A review of the literature indicates that regular drinking of mountain tea promotes weight loss and prevents insulin resistance by lowering the level of glucose and triglycerides in the blood, as well as increasing the glycogen content in the liver. In addition, it exhibits antioxidant properties, both due to the content of naturally occurring non-enzymatic antioxidants, as well as increasing the activity of catalase in the liver [6]. *Sideritis* has been also used in the form of an ethanol extract topically on the skin, as well as an antiseptic solution after tooth extraction [5]. There are also premises with a positive effect on memory and general cognitive abilities, especially in the elderly, most likely resulting from increased cerebral blood flow and higher content of oxygenated hemoglobin in the blood, especially in the prefrontal cortex. Extract from *S. scardica* also acts as a reuptake inhibitor of monoamine neurotransmitters—serotonin, noradrenaline, and dopamine—in vitro which may result in a better mood when using by humans [7,8]. Due to the high concentration of phenolic compounds, *S. scardica* extracts exhibit dose-dependent anti-inflammatory and gastroprotective activities. Moreover, the high content of some phenolic compounds, especially certain flavonoids may result in cytotoxic activity against cancer cells [9].

The way in which phenolic compounds affect the body depends largely on their bioavailability and absorption from the intestine. Some reports indicate that high concentration of phenolics in a daily diet may influence variability of gut microbiota promoting beneficial bacteria like *Lactobacillus* and *Bifidobacterium* spp. and inhibiting the growth of pathogenic bacteria such as *Clostridium* spp. In turn, intestinal microbiota play important role in the transformation of polyphenols into bioactive and bioavailable compounds [10].

In view of the growing interest in functional food and enriching the diet with natural compounds with health-promoting potential, it seems reasonable to analyze and comprehensively summarize the knowledge about bioavailability and mechanism of action of major biologically active compounds of *S. scardica.* Because of the limited amount of available scientific literature on the species *S. scardica*, we have expanded our analysis to include other related species from the genus *Sideritis*. Based on comprehensive analysis we assume *S. scardica* to be potentially effective in preventing metabolic disturbances resulted from overweight and obesity (including insulin resistance, hypercholesterolemia, and hyperlipidemia) with simultaneous positive effects on mental performance, especially in the elderly.

## 2. Phytochemical Profile of *Sideritis scardica* and Other *Sideritis* Species

To protect their own tissues, plants developed an efficient antioxidant system. Among non-enzymatic antioxidants, phenolic compounds deserve special attention. To this day, a diet rich in polyphenols derived mainly from fruits, vegetables, and beverages such as tea, coffee, or wine is valued in the prevention of premature aging, cardiovascular diseases, and even cancer.

Qualitative analysis of polyphenolic compounds from several related *Sideritis* species (*S. scardica*, *S. raeseri*, *S. taurica*, *S. syriaca*, and *S. perfoliata*) from the Balkan Penisula revealed that all the taxa produce a similar set of polyphenols (Table 1). Methanol extracts from the above-mentioned *Sideritis* species were characterized by the presence of mainly phenylethanoid glycosides (echinacoside, lavandulifolioside, verbascoside, forsythoside A, isoverbascoside, samioside, allysonoside, and leucoseptoside A) and flavonoid acetylglycosides (3′-*O*-methylhypolaetin 7-O-[6‴-*O*-acetyl]-allosyl(1→2)glucoside and 4′-*O*-methylhypolaetin 7-O-[6‴-*O*-acetyl]-allosyl-(1→2)- [6″-*O*-acetyl]-glucoside), which accounted for 90% of all phenolic compounds [11].

Apart from flavonoid glycosides, *Sideritis* species (*S*. *scardica* and *S*. *raeseri*) also contain 5,7-hydroxyflavones (apigenin and luteolin) and 8-hydroxyflavones (isoscutellarein (8-OH apigenin) and hypolaetin (8-OH luteolin) and their derivatives) [13]. The presence of 8-hydroxyflavones such as isoscutellarein and hypolaetin and their methoxy derivatives is very characteristic for *Sideritis* plants and was confirmed in later studies [3]. Furthermore, in *Sideritis* extracts hydroxycinnamic acid derivatives were detected: 3-*O*-caffeoylquinic acid and 5-*O*-caffeoylquinic acid (main representative of chlorogenic acids), *p*-coumaric acid 4-*O*-glucoside, and feruloylquinic acid [11,13]. Later studies confirmed that plants belonging to the genus *Sideritis* (samples of *S. scardica* and *S. raeseri*) collected from the Balkans countries are similar in chemical composition [12]. Similar polyphenolic pattern indicates that all these species are very closely related and may exert the same health benefits on human.

Although the genus *Sideritis* belongs to the *Lamiaceae* family, it is not very rich in essential oils. Despite the low concentration of essential oils, the infusion of *S. scardica* has a very pleasant aroma and it is used as a refreshing herbal tea, sometimes with honey and lemon juice [15]. According to the review of Todorova et al. [5] the chemical composition of essential oils extracted from *S. scardica* differs significantly depending on the location of obtaining plants. These differences may result from soil and climate conditions, as well as parts of the plants from which oils were extracted (flowering tops, leaves or whole aerial parts). Notwithstanding the quantitative disparity in the *S. scardica* essential oils dominate monoterpenes, sesquiterpenes, and diterpenoids [5].

**Figure 1:**
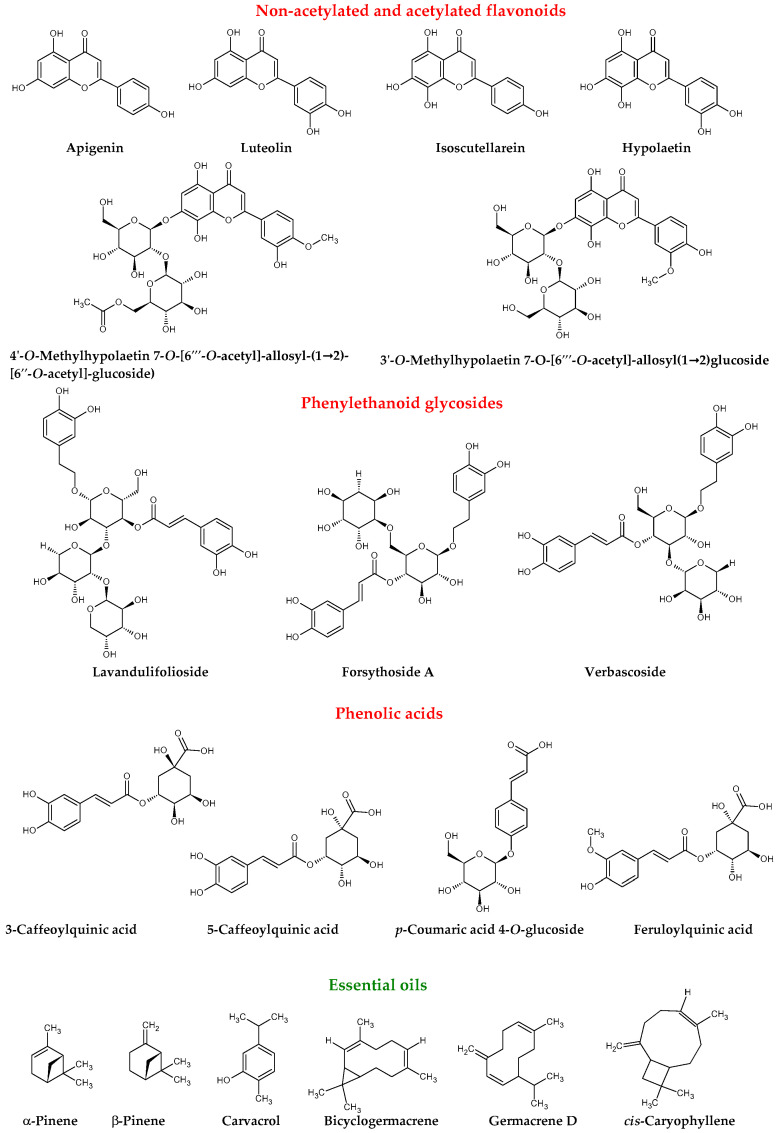
Main polyphenolic compounds and essential oils of *Sideritis scardica* [18].

The major components of water-distilled essential oil from plants collected in European part of Turkey were β-pinene (17.9%), carvacrol (14.8%), and α-pinene (7.3%) [16]. In one of the recent researches, other authors examined two different samples of *S. scardica* organically cultivated in two regions of Greece and noticed that main constituents of essential oils were as follows; α-pinene (8.2–17.8%), β-pinene (12.8–13.1%), *cis*-caryophyllene (6.6–7.6%), bicyclogermacrene (6.6–7.1%), and germacrene D (2.2–6.6%). Authors suggested that factors like altitude and cultivation methods play a significant role in the chemical composition of essential oils and may be responsible for quantitative differences between these two populations of *S. scardica* plants [17]. Chemical structures of main polyphenolic compounds and essential oils of *S. scardica* are presented in Figure 1.

Probably the antimicrobial properties of the *Sideritis* extracts result from the presence of monoterpenes such as α-pinene, β-pinene, and carvacrol with proven antibacterial and antifungal activity. Some essential oils extracted from different species from *Lamiaceae* family are used in medical products and cosmetics [19,20]. As the genus *Sideritis* belongs to the *Lamiaceae* family, we may suspect similar properties of its essential oil components. As *Sideritis* plants are most often used in the traditional form of an aqueous infusion, it is worth mentioning that brewing conditions affect the content of phenolic compounds and antioxidant activity of the *Sideritis* tea infusion. Research conducted by Irakli et al. [21] suggests that the optimal temperature ranged between 87.5 and 99.8 °C with infusion time no longer than 10 min. The highest content of biologically active compounds, dominated by chlorogenic acid, was detected in leaves infusions. In addition to polyphenolic compounds, minerals were detected in the concentration as follows, K > P > Ca > Mg > Na > Mn > Fe > Cu > Zn.

## 3. Beneficial Impact of Compounds Present in *Sideritis* Plants on Mental Health

As previously noted, the basic bioactive compounds in mountain tea seem to be phenylethanoid glycosides, flavonoid acetylglycosides (mainly apigenin, luteolin, isoscutellarein and hypolaetin derivatives), and hydroxycinnamic acid derivatives, mainly ferulic acid and caffeic acid [3,11,13] with diverse biological activities which are summarized in Table 2. This is an important finding, considering that phenolic compounds found in *S. scardica* extracts have been found to have not only antioxidant, but also anti-inflammatory and neuroprotective properties, which was confirmed in an animal model [22,23,24].

The potential bioactivity of the extracts from *Sideritis* spices are summarized in Table 3. Extracts from *S. scardica* rich in flavones and phenolic acids show the activity of monoamine reuptake inhibitors (that inhibits reuptake of a monoamine neurotransmitters: serotonin, noradrenaline and dopamine, from the synapse into the pre-synaptic neuron) [8]. Apigenin alone is able to interact with monoamine transporters [7], and is supposed to be a ligand for benzodiazepine receptor. The latter activity may be the basis of anxiolytic effect, which has been confirmed in studies in an animal model in mice [43] and may underlie the improvement of mood in human. A pilot study conducted by Behrendt et al. [72] on a group of 64 healthy adults aged 25–60 allowed observation of cognitive improvement after six weeks of supplementation with extract of *S. scardica* 330 mg in combination with supplementation of B vitamins: vitamin B1 (0.55 mg), vitamin B6 (0.7 mg), vitamin B12 (1.25 µg), and folic acid (100 µg) twice a day (morning and evening; after meals). The results proved that the supplementation of *S. scardica* extracts and selected vitamins can shorten response time and alleviate stress-induced cognitive impairment (executive functioning in terms of working memory, cognitive flexibility and controlled behavioral inhibition after a six-week intake period).

Despite the presence of many drugs on the market, patients, especially those requiring long-term therapy, prefer herbal medicines of natural origin. The activity of *S. scardica* extracts as triple monoamine reuptake inhibitors gives hope for its use in phytotherapy of neurobehavioral disorders associated with disturbance in monoaminergic neurotransmission. Aqueous or ethanolic *S. scardica* extract affecting all three neurotransmitters—serotonin, dopamine, and noradrenaline—may be a potential candidate for a natural drug in the treatment of i.a. depression, anxiety disorders, attention-deficit hyperactivity disorder (ADHD), neuropathic pain, alcohol abuse and obesity, or even neurodegenerative diseases. Due to the above-discussed properties, the pharmacological activity of the whole extracts as well as their single components needs further investigation in vivo.

Other plant species of the genus *Sideritis* also seem to have such properties. Vasilopoulou et al. [73] investigated the effect of drinking *Sideritis clandestina* subsp. *clandestina* infusion for 6 weeks on the behavior of adult mice. Mice were submitted to the open-field behavioral thigmotaxis test to check if they walk close to walls when exploring an open space (such a behavior is a well-established indicator of animal fear). Researchers confirmed the effect of *Sideritis* herbal tea on reducing anxiety in mice. In addition to anxiolytic effect, it was showed that drinking herbal tea from *Sideritis* resulted in inhibition of lipid peroxidation, measured by a significant decrease in MDA (malondialdehyde/lipid peroxidation marker) concentration in cerebellum and midbrain but not in cerabral cortex. Equally important, significant increase in GSH (reduced glutathione) concentration in cerebellum and midbrain has been observed. As MDA is the final product of lipid peroxidation, whereas GSH is a tripeptide which acts as a free radical scavenger and detoxifying agent, such results confirm the antioxidant effect and support neuroprotective role of this herbal tea. Melittoside derivatives, quinic acid derivatives, and apigenin as the main compounds identified in *Sideritis clandestina* aqueous extract may be responsible for such properties. There are scientific reports of anti-inflammatory [59], neuroprotective, and neurotrophic activity of quinic acid derivatives [60,61], while, as mentioned previously, apigenin is thought to exert anxiolytic activity [43] and to play as neuroprotective agent [44,45]. Some reports indicate that apigenin alone also support antioxidant system, leading to increased expression of enzymes: GSH-synthase, catalase, and superoxide dismutase [46,47,48,84]. All these premises make *Sideritis* a potential candidate to support the treatment of neurodegenerative diseases but pharmacological properties of particular biological active compounds are still not clear.

Cognitive improvement may also result from the presence of phenolic acids mainly ferulic acid and chlorogenic acid (esters of quinic and caffeic acids) in extracts of *S. scardica*. Both ferulic [85] and chlorogenic acid [62] have the ability to interact with nitric oxide (NO)—the vasodilatory mediator, which results in changes of peripheral blood flow in the animal and human models. Acutely reduced blood pressure (systolic and diastolic) was noticed in healthy adults after 400 mg of chlorogenic acid [62]. Similar results were obtained in a long time period in patients with mild hypertension who were given 140 mg of chlorogenic acid daily for 12 weeks [63]. The acute effects of chlorogenic acid, the main phenolic acid present in coffee, on cognition, is generally stronger with the presence of caffeine but both caffeine and other compounds like polyphenols different than chlorogenic acid present in coffee may be responsible for this effect. The advantage of *Sideritis* extracts in comparison to coffee is a lack of caffeine which is not always desirable in a diet.

Wightman et al. [75] conducted a double-blind, placebo control trial on a group of 155 healthy, older adults (50–70-year old) who were given *S. scardica* extract at a dose of 475 or 950 mg daily for 28 days. As reference, placebo and active control groups were created. The active control group was given 240 mg of *Ginkgo biloba* daily. Supplementation of *S. scardica* in a dose of 950 mg significantly reduced anxiety after 28 days when comparing to placebo and *Ginkgo biloba* extract. The higher dose of *S. scardica* extract also improved accuracy on the picture recognition task and both doses significantly faster speed of attention after acute administration (first dose) and after long time supplementation. What is important both doses of *S. scardica* extract increased cerebral blood flow(CBF) measured by the increased concentration of oxygenated hemoglobin (HbO) and oxygen saturation (Ox%) in the prefrontal cortex during completion of cognitively demanding tasks after acute administration. The increase of total and deoxygenated hemoglobin was also observed even after first administration of higher dose of *Sideritis* extract. It seems most probable that synergistic and multiple action of polyphenols resulted in inhibition of the reuptake of monoamine neurotransmitters and increase cerebral blood flow is responsible for anxiolytic, cognitive improving, and neuroprotective properties.

The potential effectiveness of *Sideritis* extracts in preventing the development of neurodegenerative diseases has also been tested in animal models. Hofrichter et al. [76] confirmed prevention against amyloid-β-induced memory impairment by herbal extracts of *S. scardica* and *Sideritis euboea* in transgenic mouse model of Alzheimer’s Disease, especially when extracts from both species were used in combination. The authors noticed decreased number and size of amyloid-β plaques as well as an improved cognition in animal model. Similar results were provided by studies using transgenic *Caenorhabditis elegans* expressing amyloid-β. In worms treated with *S. scardica* extract, a reduced number of amyloid-β plaques in the head region and lower amyloid-β toxicity were observed [86]. As research on animal model confirmed that apigenin exhibits protective effect against amyloid-β-induced neurotoxicity [23,44,49], we conclude that apigenin is most likely responsible for neuroprotective effect also in human but the mechanism is unclear.

## 4. Anti-Inflammatory and Antimicrobial Properties of *Sideritis* Plants

In addition to beneficial effects on the nervous system, mountain tea is traditionally drunk after meals to improve digestion and to speed up metabolism. In folk medicine it is also known for its anti-inflammatory and antiseptic properties. The study of Tadić et al. [77] confirmed significant anti-inflammatory activity of *S. scardica* ethanolic extract comparable with a nonsteroidal anti-inflammatory drug indomethacine. In addition, some studies reported the antimicrobial activity of *S. scardica* extracts against several common Gram-positive (*Staphylococcus epidermidis*, *Micrococcus luteus*, *Staphylococcus aureus*, *methicillin-resistant Staphylococcus aureus*, *Streptococcus pyogenes*, *Streptococcus canis*, *Moraxella catarrhalis*, *Corynebacterium pseudotuberculosis*, and *Enterococcus faecalis*) and Gram-negative bacteria (*Escherichia coli*, *Klebsiella pneumoniae*, *Pseudomonas aeruginosa*, *Pasteurella multocida*, and *Haemophilus* sp.) as well as the yeast *Candida albicans* [77]. The strongest activity of extracts was determined against *S. epidermidis*, *M. luteus*, *E. coli*, *P. aeruginosa*, *Corynebacterium pseudotuberculosis*, Pasteurella multocida, and Haemophilus sp. Worth mentioning is the antimicrobial activity which was similar for all tested extracts regardless on the extraction methods. *Similarly*, essential oil obtained from *S. scardica* by hydrodistillation most strongly inhibited the growth of *Corynebacterium pseudotuberculosis* and *Haemophylus* sp. According to authors, the combination of different types of terpenoids (especially monoterpenes and diterpenes) and fatty acids and their esters may be responsible for such antimicrobial activity. According to the study performed by Sagdic et al. [79], 10% methanolic extracts of *Sideritis ozturkii* and *Sideritis caesarea* also exhibit strong antimicrobial activity inhibiting the growth of *Aeromonas hydrophila*, *Bacillus cereus*, *Bacillus subtilis*, *Bacillus subtilis var. niger*, *Bacillus brevis*, *Escherichia coli*, *Klebsiella pneumoniae*, *Morganella morganii*, *Mycobacterium smegmatis*, *Proteus mirabilis*, *Pseudomonas aeruginosa*, *Staphylococcus aureus*, and *Yersinia enterocolitica*, and antifungal activity against *Candida albicans* and *Saccharomyces cerevisiae.*

Essential oils obtained from *S. curvidens* and *S. lanata* by hydrodistillation were identified as effective antimicrobial agents against Gram-positive bacteria, especially methicillin-resistant strain of *Staphylococcus aureus* (MRSA) and oxacillin-resistant coagulase negative *Staphylococcus epidermidis* [80].

Similarly essential oils distilled from five taxa of *Sideritis* growing in Greece belonging to the species *S. clandestina*, *S. euboea*, *S. romana*, and *S. lanata* significantly inhibited growth of Gram-positive bacteria, where *S. aureus* seems to be the most sensitive to all the tested oils [81].

Such properties of *Sideritis* plant extracts and essential oils suggest that they can be potentially used as a natural antioxidant and antimicrobial agents in food preservation. In addition, considering their effectiveness against antibiotic-resistant bacteria strains, they may be used as novel antimicrobial agents in human infections therapies.

## 5. Effect of *Sideritis scardica* and Other *Sideritis* Plants on Blood and Liver Parameters

In addition to antimicrobial activity, biological active compounds of *Sideritis* seem to have a positive effect on carbohydrate and fat metabolism. In a rat menopause model (ovariectomized females), it was observed that rats given *S. scardica* extract had lower blood triglyceride levels, reduced fasting glucose levels, and lower glucose peaks following oral glucose challenge. Furthermore, an increase in glycogen content and thiol groups concentration in the liver, as well as an increase in the enzymatic activity of catalase compared to the control group (ovariectomized rats that were not given the extract) were observed. Probably such an effect of dietary supplementation with *S. scardica* extract resulted in alleviation of metabolic disturbance what was associated with the activation of AMP-activated protein kinase in liver cells (AMPK) [6].

Protein kinase activated by AMP (AMPK) is the main sensor of the metabolic state, protects the metabolic balance, both at the cellular and the whole organism level. AMPK is activated as a result of increasing cellular AMP concentration, with a decrease in ATP during energy deficiency, among others caused by hunger or intense training. Activated AMPK leads to the activation of ATP-producing catabolic pathways with simultaneous inhibition of energy-consuming anabolic processes. A prolonged decrease in AMPK activity, caused by an excessively caloric diet and lack of physical activity, is associated with the emergence of insulin resistance, type 2 diabetes, and the development of other metabolic disorders. Changes in AMPK activity are also observed in inflammation [87]. There are reports in the literature of frequent co-occurrence of obesity, type 2 diabetes, and cancer, which may be caused by disturbance in the proper functioning of AMPK kinase.

To date, several AMPK activating substances have been discovered and tested in animal models of type 2 diabetes showing promising results. Among them, naturally occurring alkaloids and polyphenols, as well as synthetic compounds, e.g., nucleotide analogues have been found [88]. However, the practical use of AMPK activators in the treatment of insulin resistance requires extensive knowledge on the mechanism of action and determining whether specific activation of AMPK will be safe for maintenance of long time homeostasis.

In 2006, Zang et al. [82] confirmed that hepatic APMK inactivation is crucial in the pathogenesis of hyperlipidemia in diabetes. The study revealed that polyphenols, including resveratrol (a major polyphenolic compound in red wine) and apigenin, which is also present in *S. scardica*, increased the activity of AMPK in human HepG2 hepatocytes in vitro. It is worth mentioning that apigenin was 200 times more effective in activation of AMPK than metformin—the most commonly used antihyperglycemic drug in the treatment of type 2 diabetes.

Banerjee et al. [89], while conducting research in mice, observed an increase in hepatic AMPK activity as a result of its phosphorylation after oral administration of green tea extract at a dose of 50 and 100 mg/kg body weight (b.w.) as opposed to no such effect after administration of black tea extract, even at a dose of 250 mg/kg b.w. Most likely, this effect is a result of the properties of catechins—flavonoids abundantly found in green tea. Similarly, chlorogenic acid is able to activate AMPK, resulting in suppression of glucose and fatty acids biosynthesis in liver [90].

Kassi et al. [83] conducted a double-blind, placebo control trial on a group of 47 healthy adults. The study group comprised 26 participants who supplemented the diet with 300 mg *S. euboea* aqueous extract daily for 30 days. The results confirmed the beneficial impact of *S. euboea* on total cholesterol level, which decreased significantly, but any significant effects on other lipid parameters and inflammatory markers were not observed. Surprisingly, enriching the diet with *S. euboea* ameliorated insulinemia, but only in women. As the level of adiponectin is generally higher in women than in men, it is possible that not only potent activation of AMPK, but also the upregulation of adiponectin may be responsible for the differences between genders. A thorough understanding of the mechanism of action requires, however, extensive research conducted on a large population of both healthy adults as well as obese ones with hypercholesterolemia, hyperlipidemia, and diabetes.

The exact mechanism by which polyphenolic compounds activate AMPK is not clear. It is supposed that polyphenols interact with mitochondrial respiratory chain what results in inhibition of mitochondrial ATP production.

## 6. Interactions of Bioactive Compounds from *Sideritis scardica* with the Intestinal Microflora

The biological properties of phenolic compounds are often evaluated on cell or tissue cultures using polyphenols in their original form in which they are present in food of plant origin. As it is known, the interaction between polyphenols and intestinal microbiota may be bidirectional. Phenolic compounds may act in the gut microbiota promoting the growth of beneficial bacteria and inhibiting the growth of pathogenic bacteria. Moreover, the gut microbiota play a key role in the biotransformation and the biological activities of polyphenols. However, polyphenols are extensively metabolized not only by the intestinal microbiota but then at least in the liver. It is therefore necessary to understand how they affect the intestinal microflora and how they are metabolized.

Polyphenols of plant origin in the human diet usually occur in the form of glycosides, and, as they do not belong to the macro- and micronutrients, are not primarily absorbed from the intestines into the blood. Bioavailability of polyphenols mostly depends on their complexity and molecular weight. Only phenolic compounds of low molecular weight—simple phenols and dimers (up to 10% of total intake)—are absorbed directly in the small intestine after deglycosylation. The remaining high molecular weight polyphenols (mainly proanthocyanidins—condensed tannins) reach the large intestine, where undergo successive enzymatic transformations due to the presence of gut microbiota [10,91].

Petreska Stanoeva and Stefova [92] performed a feeding study with 10 human volunteers concerning metabolism of polyphenols found in *S. scardica* decoction. The urinary extraction of hydroxycinnamic acids, phenylethanoid glycosides, and flavonoids metabolites was sampled up to 24 h after consumption of *S. scardica* decoction. In this study, 63 different metabolites of flavonoids (hypolaetin, isoscutellaerin, and apigenin derivatives) and phenolic acids were found in the urine, in the form of glucuronidated, sulfated, and native compounds. The study showed that despite the fact that flavonoids initially present in the *Sideritis* decoction have very complex structure (aglycone-diglucoside-(mono- or di-) acetyl), these compounds were transformed into glucuronides, sulfates, and *O*-methylated forms and excreted in urine (Table 4). Some authors stated that hydroxyl groups at the 5-, 7-, and 4′-positions of flavonoids are important structural features for optimal degradation of flavonoids by human intestinal microflora [11,92,93]. Contrary to decoction, in which only 4 hydroxycinnamic acid derivatives were detected (3-caffeoylquinic acid, 5-caffeoylquinic acid, *p*-coumaric acid 4-*O*-glucoside, and feruloylquinic acid), as many as 32 different metabolites were found in urine (quinic acid derivatives, caffeic acid derivatives, caffeoylquinic acid derivatives, ferulic acid derivatives, dimethylferulic acid derivatives, feruloylquinic acid derivatives, and coumaric acid derivatives) as glucuronide; mono-, di-, or tri-sulfate; and glucuronide sulfate. Only caffeic acid was detected in the free form in urine. Despite the fact that phenylethanoid glycosides are the group of polyphenolic compounds present in the highest concentration in *Sideritis* infusions and decoction, no metabolites of phenylethanoid glycosides in urine were found. Authors speculated that hydroxycinnamic acid metabolites in urine may be derived not only from hydroxycinnamic acids naturally present in *Sideritis* but also from phenylethanoid glycosides. Another possible explanation is the low absorption of this group of compounds in the intestines. This is in line with the results of other studies according to which bioavailability of phenylethanoid glycosides after oral administration is relatively low [94,95]. On the other hand study conducted by Wang et al. [96] revealed that phenylethanoid glycosides go through extensive metabolism by intestinal bacteria what may change them in more bioavailable forms but still with potent antioxidant activity. Metabolic transformations of phenylethanoid glycosides mainly include enzymatic deglycosylation, reduction, hydration, hydroxylation, acetylation, methylation, and sulfate conjugation.

Phenolic compounds already have a positive effect in the intestines. As the highest concentration of polyphenols is found in the intestinal lumen, they can already protect the intestinal mucosa from oxidative stress and carcinogens. Tannins and related complex polyphenols from red wine administered to rats orally were shown to protect chemically induced oxidative DNA damage of hepatocytes [114]. A similar protective effect was observed in rats treated with black tea polyphenols that reduced chemically induced oxidative DNA damage in colon mucosal cells [115]. Such properties of polyphenols are very promising in protection against oxidative damage of intestinal and liver cells, as well as in preventing gastrointestinal inflammation and carcinogenesis. Tadić et al. [9] confirmed the gastroprotective activity of *S. scardica* extracts using rat model of an acute ethanol-induced gastric mucosa damage. The protective effect on the gastric mucosa was comparable to antiulcer drug ranitidine.

## 7. Conclusions

Understanding the multifaceted potential and mechanism of action of polyphenol-rich *S. scardica* extracts and finding an analogy in the biological role of compounds naturally found in other plants, creates a wide field for developing an effective composition of an innovative functional foods and nutraceuticals to combat the common effects of overweight and obesity. A growing number of researches indicate a positive influence of *Sideritis* on gut microflora, digestive system, prevention of insulin resistance and hiperlipidemia. Additionally, strong antioxidant and anti-inflammatory activity support health promoting effects, as free radicals are thought to be responsible for numerous pathologies like chronic inflammation and oxidative damage to cellular structures leading, among others, to the development of cancer diseases. A positive effect of supplementation the diet with *Sideritis* extracts on general cognitive abilities has been reported as well. The advantage of functional foods and nutraceuticals based on *S. scardica* would be a simultaneous beneficial effect on lipid parameters and cardiovascular risk factors as well as a positive influence on memory and mood improvement, especially in the older adults. Due to the insufficient data from human studies, further research is needed, especially clinical studies on a large population. It is also a need to expand research on metabolism of polyphenolic compounds present in *S. scardica*.

## Figures and Tables

**Table 1:** Main phenolic compounds found in *Sideritis* species.

Compound	Ref.
***Phenylethanoid glycosides***	
Lavandulifolioside	[3,11,12]
Verbascoside	[3,11,12,13,14]
Forsythoside A	[3,11,12,13]
Echinacoside	[3,11,12,13,14]
Isoverbascoside	[3,11,12,14]
Samioside	[3,11,12,13,14]
Leucoseptoside A	[3,11,12,13,14]
Allysonoside	[3,11,13,14]
Martynoside	[14]
***Flavonoid glycosides***	
3′-*O*-Methylhypolaetin 7-*O*-[6‴-*O*-acetyl]-allosyl(1→2)glucoside	[3,11,12,13]
4′-*O*-Methylhypolaetin 7-*O*-[6‴-*O*-acetyl]-allosyl-(1→2)- [6″-*O*-acetyl]-glucoside	[3,11,12,13,14]
4′-*O*-Methylisoscutellarein 7-*O*-[6″-*O*-acetyl]-allosyl-(1→2)glucoside	[11,12,13]
4′-*O*-Methylisoscutellarein 7-*O*-allosyl(1→2)glucoside	[3,11,12,13]
3′-*O*-Methylhypolaetin 7-*O*-allosyl(1→2)glucoside	[3,12,13]
4′-*O*-Methylisoscutellarein 7-*O*-[6‴-*O*-acetyl]-allosyl-(1→2)-[6″-*O*-acetyl]-glucoside	[3,11,13]
Isoscutellarein 7-*O*-[6‴-*O*-acetyl]-allosyl(1→2)glucoside	[3,11,13]
Isoscutellarein 7-*O*-[6‴-*O*-acetyl]-allosyl-(1→2)-[6″-*O*-acetyl]-glucoside	[13]
Isoscutellarein 7-*O*-allosyl(1→2)glucoside	[3,11,12,13]
Hypolaetin 7-*O*-[6‴-*O*-acetyl]-allosyl(1→2)glucoside	[11,12,13,14]
Hypolaetin 7-*O*-allosyl-(1→2)-[6″-*O*-acetyl]glucoside	[3,11,12,14]
Apigenin 7-(4″-*p*-coumaroylglucoside)	[3,11,14]
Apigenin 7-(6″-*p*-coumaroylglucoside)	[3,11,14]
Apigenin 7-*O*-allosyl(1→2)glucoside	[11,12,14]
Apigenin 7-*O*-[6″-*O*-acetyl]-allosyl(1→2)glucoside	[3,11,12,13,14]
Luteolin 7-*O*-allosyl(1→2)glucoside	[13]
Luteolin 7-*O*-allosyl-(1→2)-[6″-*O*-acetyl]-glucoside	[11,12,14]
Luteolin 7-O-[6‴-O-acetyl]-allosyl(1→2)glucoside	[11,12]
Luteolin 7-O-[6‴-*O*-acetyl]-allosyl-(1→2)-[6″-*O*-acetyl]-glucoside	[11,12,14]
Hypolaetin 7-*O*-allosyl(1→2)glucoside	[3,11,13]
Apigenin 7-*O*-glucoside	[3,11,14]
Chryseriol 7-*O*-[6‴-*O*-acetyl]-allosyl(1→2)glucoside	[3,11]
***Phenolic acids***	
3-Caffeoylquinic acid	[13]
5-Caffeoylquinic acid	[3,11,12,13,14]
6-*O*-Caffeoyl-glucose	[14]
*p*-Coumaric acid 4-*O*-glucoside	[3,11]
Feruloylquinic acid	[3,11,12,13,14]

**Table 2:** Biological activity of most common *Sideritis scardica* phenolic compounds.

Polyphenols	Biological Activity	Ref.
*Phenylethanoid glycosides*		
Lavandulifolioside	antiarrhythmic effect peroxylipid formation inhibitor anti-inflammatory activity	[25,26,27]
Verbascoside	antioxidant activity anti-inflammatory activity prevention red blood cell from free radical damage tyrosinase and/or melanin production inhibition activity	[14,28,29,30,31,32,33,34,35]
Forsythoside	anti-inflammatory activity antibacterial activity inhibitory of cAMP-phosphodiesterase in vitro 5-HETE formation inhibitor	[26,27]
	antioxidant activity anti-inflammatory activity tyrosinase and/or melanin production inhibition activity	[14,27,29,35]
*Flavonoids*		
Apigenin	antioxidant activity anti-inflammatory effect cytotoxicity to cancer cells promoting apoptosis of cancer cells anxiolytic effect memory improvement neuroprotective effect, protective effect against amyloid-β-neurotoxicity	[9,36,37,38,39,40,41,42,43,44,45,46,47,48,49] [23,24,44,45,49]
Luteolin	antioxidant and anti-inflammatory activities cytotoxicity to cancer cells promoting apoptosis of cancer cells neuroprotective effect	[9,22,23,50,51,52,53,54,55]
Isoscutellarein	moderate to weak cytotoxicity to cancer cells	[56]
Hypolaetin	anti-inflammatory activity gastric protection (increase in mucus production) anti-ulcer activity	[57,58]
*Phenolic acids*		
Caffeoylquinic acid	antioxidant activity reduced blood pressure neuroprotective effect	[59,60,61,62,63,64]
*p*-Coumaric acid 4-*O*-glucoside	antioxidant activity cytotoxicity to cancer cells promoting apoptosis of cancer cells neuroprotective effect cytotoxicity to cancer cells	[64,65,66,67,68,69,70]
Feruloylquinic acid	antioxidant activity hepatoprotective activity anti-proliferative activity	[35,71]

**Table 3:** Biological properties of *Sideritis* plant extracts.

Biological Function	Extract	Ref.
Antioxidant activity	*S. perfoliata* air-dried aerial parts extract, *S. clandestina* aqueous extract	[29,73,74]
Anxiolytic, cognitive improving and neuroprotective properties	*S. scardica* extract (aqueous or ethanolic), *S. euboea* extract, *S. clandestina* infusion	[72,73,75,76]
Inhibition of lipid peroxidation	herbal tea from *Sideritis*	[73]
Anti-inflammatory activity	*S. scardica* ethanol, diethyl ether, ethyl acetate, and N-butanol extracts	[77,78]
Antimicrobial properties	*S. scardica* extract, *S. ozturkii* and *S. caesarea* methanolic extracts, essential oils from *S. curvidens*, *S. lanata. S. clandestina*, *S. euboea*, and *S. romana*	[77,78,79,80,81]
Gastroprotective effect	*S. scardica* ethanol, diethyl ether, ethyl acetate, and N-butanol extracts	[9]
Anti-obesity and antidiabetic properties	*S. scardica* extract, *S. euboea* aqueous extract	[6,82,83]
Chemopreventive activity	*S. scardica* diethyl ether extract	[41,42]

**Table 4:** Summary of the main metabolites and colonic catabolites of the most common *Sideritis scardica* phenolic compounds.

Polyphenol Class	Compounds	Phase I and II Possible Metabolites	Potential Microorganisms Involved	Microbial Biotransformation	Possible Gut Microbial Metabolites	Ref.
Flavonoids	Hypolaetin	Hypolaetin sulfate, glucuronide, diglucuronide, and glucuronide-sulfate	Human and mice fecal flora	Hydrolysis by gut microflora into their aglycones	Hypolaetin, isoscutellarein, and methylhypolaetin	[92,97]
	Methylhypolaetin	Methylhypolaetin sulfate, glucuronide, and glucuronide + pentose				
	Isoscutellarein	Isoscutellarein sulfate, glucuronide, and glucuronide-sulfate				
	Methylisoscutellarein	Isoscutellarein disulfate, glucuronide, and diglucuronide				
	Apigenin	Apigenin disulfate, glucuronide, diglucuronide, and glucuronide-sulfate	Human and rats fecal flora	Hydrolysis by gut microflora to simple phenolic acids	3-(4-Hydroxyphenyl)propionic acid, 3-(3-hydroxyphenyl)propionic acid, 3-(3,4-dihydroxyphenyl)propionic acid, phenylacetic acid, 4-hydroxycinnamic acid, phloretin	[92,96,98,99,100]
	Luteolin	Luteolin glucuronide and sulfate, o-methyl luteolin (diosmetin or chrysoeryol)	Human and rats fecal flora	Hydrolysis by gut microflora to simple phenolic acids	3-(3,4-dihydroxyphenyl)propionic acid, 3-(4-hydroxyphenyl)propionic acid and 4-hydroxycinnamic acid, phloretin, eriodictyol, and phloroglucinol	[92,99,101,102,103]
Phenylethanoid glycosides	Verbascoside (acteoside)	Methyl acteoside, dimethyl acteoside, methyl acteosideglucuronide, dimethyl acteosideglucuronide, caffeic acid sulfate and glucuronide, methyl caffeic acid sulfate, hydroxytyrosolsulfate and glucuronide, homovanillic alcohol sulfate and glucuronide, homovanillin glucuronide, homovanillic acid, homovanillic acid sulfate and glucuronide, ferulic acid, ferulic acid glucuronide, and homoprotocatechuic acid	Human and rats fecal flora	Deglycosylation, de-rhamnose, de-HT, de-caffeoyl, deacetylation, reduction, acetylation, and sulfate conjugation	Caffeic acid,3-hydroxyphenylpropionic acid and hydroxytyrosol	[104,105,106,107,108,109]
Phenolic acids	Caffeoylquinic acid	Caffeoylquinic acid sulfate, disulfate, and glucuronide, caffeic acid glucuronide-sulfate, dimethylcaffeic acid glucuronide, quinic acid trisulfate, glucuronide, and glucuronide-sulfate	Human fecal flora	Deesterification, reduction of a double bond, dihydroxylation and futher *β*-oxidation by gut microflora to simple phenolic acids	Dihydrocaffeic acid, dihydro-isoferulic acid, 3-hydroxyphenylpropionic acid and benzoic acid	[92,105,106,107,108,110]
	Feruloylquinic acid	Ferulic acid sulfate, glucuronide, and glucuronide-sulfate, feruloylquinic acid disulfate, glucuronide, and dimethylferuloylquinic acid glucuronide			Feruloylglycine, dihydroferulic acid, and 3-(4-hydroxyphenyl)-propionic acid, benzoic acid, 3-(4-hydroxyphenyl)propionic acid, vanillin	[92,110,111,112]
	*p*-Coumaric acid 4-*O*-glucoside	Coumaric acid glucuronide			3-Hydroxyphenylpropionic acid, benzoic acid, 3-(4-hydroxyphenyl)propionic acid, vanillin	[92,112,113]
